# Mpox Hepatic and Pulmonary Lesions in HIV/Hepatitis B Virus Co-Infected Patient, France

**DOI:** 10.3201/eid3011.241331

**Published:** 2024-11

**Authors:** Ruxandra Calin, Claire Périllaud-Dubois, Stéphane Marot, Khaldoun Kerrou, Gilles Peytavin, Marwa Bachir, Anne Laure Kirch, Ludovic Lassel, Vincent Fallet, Joel Gozlan, Jean Baptiste Pain, Patricia Senet, Olivier Ferraris, Sébastien Bine, Mathieu Hubert, Olivier Schwartz, Laurence Morand-Joubert, Gilles Pialoux

**Affiliations:** Author affiliations: Sorbonne University Tenon Hospital, Paris, France (R. Calin, K. Kerrou, M. Bachir, A.L. Kirch, L. Lassel, V. Fallet, J.B. Pain, P. Senet, G. Pialoux); Sorbonne University Saint-Antoine Hospital, Paris (C. Périllaud-Dubois, J. Gozlan, L. Morand-Joubert); Sorbonne University Pitié-Salpêtrière Hospital, Paris (S. Marot); Université Paris Cité Bichat Claude Bernard Hospital, Paris (G. Peytavin); Institut de Recherche Biomédicale des Armées National Reference Center for Orthopoxviruses, Brétigny-sur-Orge, France (O. Ferraris); Directorate-General for Health, Paris (S. Bine); Université Paris Cité Institut Pasteur, Paris (M. Hubert, O. Schwartz)

**Keywords:** mpox, monkeypox virus, HIV, HIV/AIDS and other retroviruses, hepatitis B virus, HBV, liver abscess, tecovirimat, human vaccinia immune globulins, vaccinia immune globulins intravenous, VIGIV, necrotic lesions, liver, lungs, sexually transmitted infections, viruses, zoonoses, France

## Abstract

We report a case of persistent disseminated mpox evolving over >6 months in an HIV/hepatitis B virus co-infected patient in France who had <200 CD4+ cells/mm^3^, pulmonary and hepatic necrotic lesions, persistent viremia, and nasopharyngeal excretion. Clinical outcome was favorable after 90 days of tecovirimat treatment and administration of human vaccinia immunoglobulins.

By November 2023, the global mpox outbreak that began in May 2022 had resulted in >92,000 cases and 171 deaths across 116 countries (*1*). Among HIV-infected persons, prevalence was high (27%–60%); the most severe and fatal outcomes were observed in those who had advanced infections (*2*,*3*). A new mpox strain in Africa prompted the World Health Organization to declare a global emergency (*4*). Despite ongoing clinical trials, no established guidelines exist for managing severe mpox cases (*3*). We report successful management of persistent disseminated mpox having nodular liver and bilateral lung involvement in an immunosuppressed patient co-infected with HIV and hepatitis B virus (HBV).

A 49-year-old HIV/HBV–co-infected patient who identified as a man who has sex with men was hospitalized in September 2022 for disseminated necrotizing mpox skin and anal lesions. Despite a low HIV-1 virus load (54 copies/mL) under multidrug therapy, his CD4+ cell count was low (82 cells/μL), and HBV virus load was high (8.02 log IU/mL) because of poor tenofovir adherence. A 14-day tecovirimat course (Appendix Figure) improved his skin and anal lesions. However, after discontinuing treatment, new cutaneous nodular lesions appeared, and existing lesions worsened. A computed tomography (CT) scan revealed a 4-cm necrotic mass in the right upper lung lobe, nodules in the opposite lung, and perirectal and nodular liver lesions. Rehospitalized and suspected of having metastatic cancer, he also had ulceronecrotic lesions on his foot, hands, and forearm.

A positron emission tomography/CT scan showed hypermetabolic foci in the skin, rectum, lungs, and liver (Figure, panel A). Liver magnetic resonance imaging (MRI) (Figure, panel B) showed multiple 1–2 cm abscesses across all lobes. Brain MRI and cardiac ultrasound results were unremarkable. The foot lesion sample was positive for monkeypox virus (MPXV) by PCR (cycle threshold 13.47) (Appendix Figure). Anal and skin biopsies revealed massive necrosis; a liver biopsy showed necrotic tissue without tumor cells but had a high MPXV PCR result. Blood and nasopharyngeal swab samples tested positive for MPXV (Appendix Figure). A lung biopsy indicated the presence of pulmonary adenocarcinoma that was positive for MPXV but negative for other pathogens.

**Figure:**
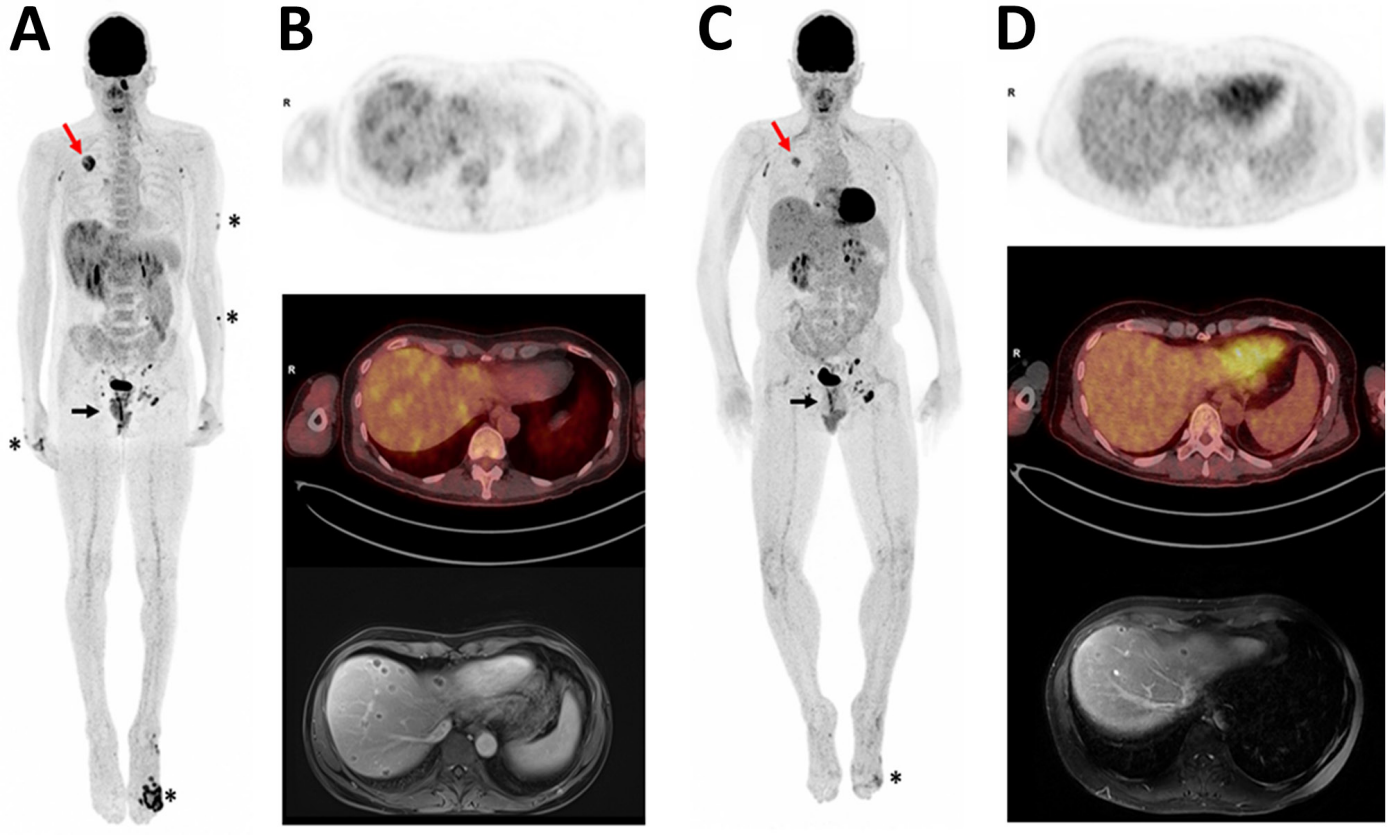
Positron emission tomography/computed tomography (PET/CT) scans and magnetic resonance imaging (MRI) of HIV/hepatitis B virus co-infected patient in case study of mpox hepatic and pulmonary lesions, France. A) Whole-body ^18^F-fluorodeoxyglucose (^18^F-FDG) PET/CT 3-dimensional maximum intensity projection performed in December 2022. Asterisks indicate anterior view of skin lesions. Black arrow indicates lesions in lower rectum; maximum standard uptake value (SUVmax) = 7. Red arrow indicates tumor in upper right lung; SUVmax = 6.4. Heterogeneous hepatic metabolism and multiple small foci of uptake were observed on liver transaxial views, which showed more intense metabolic ranges in the subcapsular region (SUVmax = 3.6). A hypermetabolic contralateral apical pulmonary nodule (SUVmax = 2.7) was also observed but is not visible in this image. B) Upper image shows liver transaxial ^18^F-FDG PET, middle image is fused PET/CT, and lower image is liver MRI (axial liver acceleration volume acquisition). C) Follow-up whole-body ^18^F-FDG PET/CT 3-dimensional maximum intensity projection performed in January 2023. Anterior view indicates substantial decreases in metabolic uptake intensities in foot lesion (asterisk), rectum (black arrow), and right lung tumor (red arrow). Tumor was 36 mm versus 46 mm in December, SUVmax 3.9 versus 6.4. Decrease in left apical pulmonary nodule, 9 mm versus 11 mm, SUVmax 1.3 versus 2.7; nodule was not visible in this image. D) Follow-up images indicate substantial decrease of liver abscesses. Upper image shows liver transaxial ^18^F-FDG PET, middle image is fused PET/CT, and lower image is liver MRI (axial T1 fat suppression volumetric interpolated breath-hold examination portal).

We readministered tecovirimat on November 25, 2022, and treated a secondary skin infection. After 1 month, the skin lesions deteriorated; blood and respiratory samples remained MPXV positive. Tecovirimat plasma levels were adequate, and virus sequencing at different time points revealed no resistance-associated mutations (i.e., *F13L* gene). We administered 2 doses of 6,000 IU/kg vaccinia immune globulin intravenous (VIGIV), which led to gradual improvement, although blood remained MPXV positive for 5 weeks. We continued tecovirimat treatment for 90 days.

We measured humoral responses to MPXV by using serologic and seroneutralization assays (Table) (*5*). Neutralizing antibody (NAb) titers in serum samples without added complement decreased when lesions reappeared but increased substantially after the first VIGIV injection. Nab titers in serum samples with added complement remained consistently high. Follow-up positron emission tomography/CT scan and MRI showed reduced lung and liver lesions (Figure, panels C, D). On February 7, 2023, we performed a right lobectomy and removed a 0.7-cm adenocarcinoma in a 2.5-cm necrotic mass; lymph nodes had no metastatic cells. By March 20, the hepatic lesions regressed, and the patient fully recovered, with no relapse as of November 2023.

**Table:** MPXV antibody titers in case study of mpox hepatic and pulmonary lesions in HIV/hepatitis B virus co-infected patient, France*

Date	No. days after mpox diagnosis	MPXV E8L IgG titer,† AU/mL	MPXV NAb titer without complement‡	MPXV NAb titer with complement‡
2022 Sep 21	35	186	160	1,280
2022 Oct 17	61	>400	0	1,280
2022 Nov 16	91	>400	40	2,560
2023 Jan 6	142	>400	1,280	2,560
2023 Jan 10	146	>400	640	2,560
2023 Feb 14	181	>400	320	2,560

*AU, arbitrary unit; MPXV, monkeypox virus; NAb, neutralizing antibody. †IgG against MPXV E8L protein was measured by using the Monkeypox Virus E8L Protein Human IgG ELISA Kit (RayBiotech, https://www.raybiotech.com). All serum samples were positive and reached the upper limit of detection (400 AU/mL) by 2 months after mpox diagnosis. ‡MPXV neutralizing antibody titers were determined in serum samples with or without added complement, as previously described (*5*). Nab titers decreased in serum samples without added complement when new skins lesions appeared and older lesions deteriorated; NAbs only increased after the first injection of vaccinia immune globulins. However, Nab titers were higher when complement was added to the assay and did not decrease; we did not see an increase after the first or second vaccinia immune globulins injections. Upper limit of MPXV neutralization titer was 1:2,560.

MPXV can persist in HIV patients, causing prolonged lesions that might be fatal (*6*). However, disease persistence for >6 months is rare. Relapse after initial tecovirimat treatment is also uncommon; immune reconstitution inflammatory syndrome was considered, but it was unlikely because of the patient’s low virus load. A longer initial tecovirimat treatment course might have been beneficial (*7*,*8*). Disseminated MPXV with lung, gastrointestinal, and neurologic involvement in HIV patients has been documented (*2*,*3*,*9*), but liver nodules were unexpected. Although initial radiologic description suggested tumor lesions, biopsies confirmed MPXV was present without cancer cells. The lung adenocarcinoma, an incidental finding, was surgically managed, and the tumor tested positive for MPXV.

Tecovirimat effectiveness was limited, despite adequate plasma levels. Tecovirimat is generally considered safe, but its efficacy in treating mpox remains uncertain (*3*,*10*). Drug resistance was a concern because of the patient’s prolonged immunosuppression and MPXV replication, but virus sequencing revealed no resistance-associated mutations. Thus, we continued tecovirimat treatment for the maximum US Food and Drug Administration–approved duration of 90 days without adverse effects.

We administered VIGIV at day 31 of tecovirimat treatment, leading to gradual lesion improvement. Although lesions healed, blood was MPXV positive for 5 weeks. Nab titers (without complement) decreased before the second hospitalization, potentially reflecting clinical disease progression. The first VIGIV injection considerably increased NAb titers, but they quickly declined, suggesting that measuring Nab titers without adding complement to the serum sample might have more clinical relevance. 

In conclusion, disseminated MPXV in HIV patients with low CD4 counts can cause prolonged, severe, and potentially fatal outcomes. This case highlights the need to monitor tecovirimat concentrations and resistance mutations and underscores the potential critical role of VIGIV treatment in severe mpox cases. As mpox continues to spread, atypical manifestations and severe forms need to be acknowledged and managed, especially in at-risk patients. 

Appendix Additional information for mpox hepatic and pulmonary lesions in HIV/hepatitis B virus co-infected patient, France.
