# Supplementary material for: Mpox Hepatic and Pulmonary Lesions in HIV/Hepatitis B Virus Co-Infected Patient, France
Source: Emerg Infect Dis. 2024 Nov;30(11):2445–7. doi: 10.3201/eid3011.241331 (PMC11521180; doi:10.3201/eid3011.241331)
Supplement: Appendix — Additional information for mpox hepatic and pulmonary lesions in HIV/hepatitis B virus co-infected patient, France. [file 24-1331-Techapp-s1.pdf]

Article DOI: https://doi.org/10.3201/eid3011.241331
EID cannot ensure accessibility for supplementary materials supplied by authors. Readers who have difficulty accessing supplementary content should contact the authors for assistance.

Mpox Hepatic and Pulmonary Lesions in HIV/Hepatitis B Virus Co-Infected Patient, France

Appendix

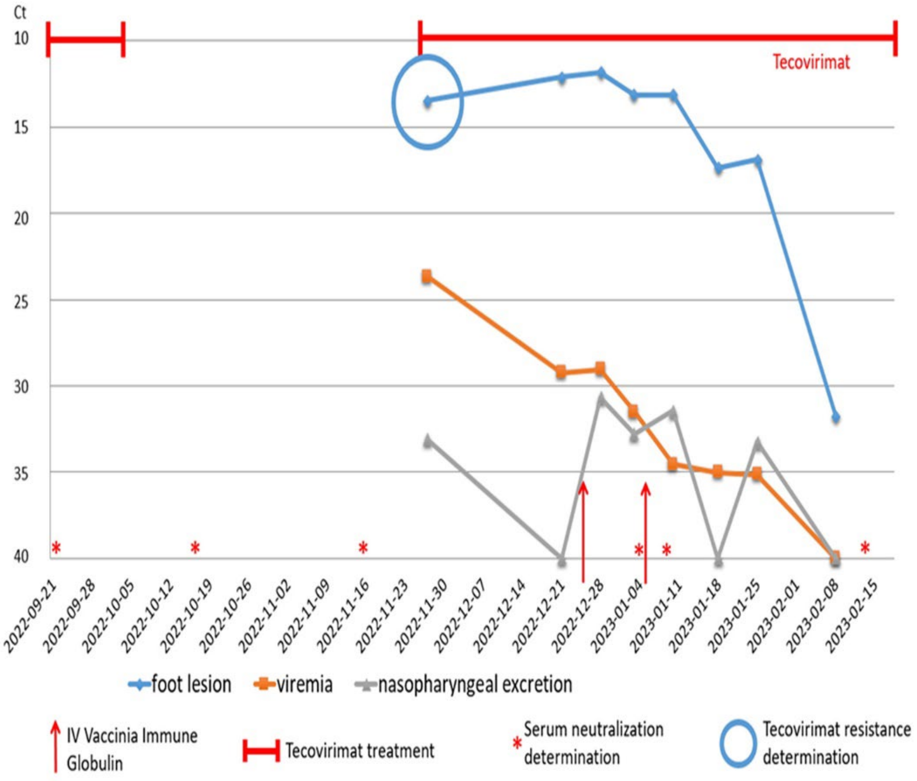

Appendix Figure. Monkeypox virus (MPXV) detected by PCR in different samples from HIV/hepatitis B virus co-infected patient during tecovirimat treatment, France. MPXV load was determined by PCR during oral treatment with 600 mg tecovirimat 2×/day along with 2 VIGIV treatments. MPXV was measured by using the RealStar Zoonotic Orthopoxvirus PCR kit (altona Diagnostics, https://www. altona-diagnostics.com). Cts were measured for blood, skin (from foot), and nasopharyngeal samples. Dates of PCR and serum neutralization assays and time course of tecovirimat treatments are indicated. Ct, cycle threshold; IV, intravenous.
